# Effectiveness of smoking cessation interventions for smokers with Crohn’s disease: a systematic review

**DOI:** 10.2144/fsoa-2022-0049

**Published:** 2023-06-01

**Authors:** Sahar Nasr, Ilyess Nsiri, Manel Ben Fredj

**Affiliations:** 1 Gastroenterology Department, University of Tunis, Tunisia; 2 Department of Preventive & Community Medicine, University of Monastir, Tunisia

**Keywords:** Crohn’s disease, inflammatory bowel disease, intervention, nursing, smoking cessation

## Abstract

**Aims:**

Smoking cessation (SC) in Crohn’s disease (CD) is widely accepted to be the most important modifiable factor to improve outcomes in these patients. We aimed in this review to provide a summary of the evidence base regarding the effectiveness of SC interventions in patients with CD.

**Materials & methods:**

The following databases were systematically searched from inception to February 2022: PubMed, Google Scholar and Cochrane Library.

**Results:**

Overall, five articles met the research criteria. Studies sample size ranged from 17 to 474 patients. At the outcome level, the abstinence rates ranged from 14.8 to 42% and was ≤25% in four studies. The three studies with control groups did not report statistically higher SC rates in the intervention groups. No predictors of interventions success were identified in this review.

**Conclusion:**

Implementation and evaluation of tailored SC interventions for CD patients must be promptly addressed in further studies.

Crohn’s disease (CD) is a chronic idiopathic inflammatory bowel disease (IBD) that can affect the entire GI tract, from the mouth to the anus. The condition was described for the first time in 1932 by Dr Burrill B. Crohn [1]. CD is more prevalent in developed countries with an annual incidence ranging from 3 to 20 cases per 100,000 and a prevalence of 130–200 per 100,000 [1–3]. CD affects men and women equally and is usually diagnosed in young active adults which puts a heavy burden on both patients and society [4]. The disease course is characterized by relapses and remissions with unpredictable flares and debilitating symptoms such as diarrhea and fatigue. Furthermore, CD requires long-term treatments with several adverse effects and in some cases surgical interventions and frequent hospital admissions which severely affects patients social, professional and psychological aspects of life [4–6]. The costs of care for patients with CD are substantial and have been steadily increasing during the last years putting a heavy burden on the health systems [4,7]. A systematic review estimated the global costs of CD in the USA and Europe combined of approximately 30 billion Euros with over half the amount due to indirect costs [8]. In addition, CD costs can sharply rise and even double or triple during flares reaching an increase of 20-fold when hospital admission is needed [9,10]. CD etiopathology is complex and determined by both genetic and environmental factors [11]. During the last decade, large-scale genome wide studies were conducted with nearly 200 CD-associated genetic loci identified [12]. Yet, this area of research resulted in no direct benefits regarding disease control and patients life quality which renewed interest in environmental factors. Tobacco smoking is indeed the most investigated environmental disease modifier in CD patients. In fact, tobacco smoking was shown to increase requirements of additional immunosuppressive agents and reduce medication efficacy and durability [13]. A systematic review and meta-analysis of 33 studies found that smokers with CD had higher risks of exacerbation of disease activity spontaneously and after surgery and were more likely to require a first and a second surgery [1]. On the other hand, all the odds of the above mentioned outcomes decreased upon smoking abstinence. In fact, smoking cessation (SC) is widely accepted to be the most important modifiable factor to improve outcomes in patients with CD [14]. A multicenter prospective study found that SC can reverse the negative disease course with quitters having comparable outcomes and prognosis to non-smokers [15]. In spite of these findings, the percentage of smokers among patients with CD remains high reaching nearly 20% in one series of cases [16]. A prospective study including smokers with CD found that approximately half of them were in the precontemplation stage which indicates no intention to quit [17]. Moreover, nearly 30% of patients with CD are unaware of the association between disease worsening and risk of reoperation and tobacco smoking [18–20]. On the other hand, SC can be very challenging which explains the low rate of individuals who manage to quit even among the ones motivated to do so [21]. To assist patients to quit, several SC interventions and programs were developed and assessed in a variety of specific populations. Yet, the inclusion of these interventions in the routine care require a thorough overview of the evidence assessing their effectiveness in CD patients.

All the above mentioned features highlight the critical need for effective and appropriate SC interventions in patients with CD. Therefore, we aimed in this review to provide a summary of the evidence base regarding the effectiveness of SC interventions in patients with CD.

## Materials & methods

This systematic review was designed and conducted in accordance with PRISMA (Preferred Reporting Items for Systematic Reviews) guidelines, to study the efficacy of SC interventions among patients with CD. The protocol of this review was submitted in International Prospective Register of Systematic Reviews (PROSPERO) and is currently being revised. Given the nature of this research study no approval by an institutional review board was necessary.

### Data sources

The following databases were systematically searched from inception to February 2022 for relevant articles published either in English or French: PubMed, Google Scholar and Cochrane Library.

### Search strategy

The search strategy was conducted according to the acronym ‘PICO’ which means the following characteristics: Population, Intervention, Comparison and Outcomes.

The target publications were collected through Medline database using a search query associating the key words mentioned in Box 1. A further research was conducted using Google Scholar (Box 2) and Cochrane Library (Box 3).

Box 1. **Search query used to collect articles indexed in Medline about from inception to March 2022**

‘(Smoking cessation)’ and (Crohn’s disease or Crohn or inflammatory bowel disease).

Box 2. **Search query used to collect articles indexed in Google Scholar**

Advanced Research/Find articles with all of the words where my words occur in the title of articles:

Smoking cessation and Crohn’s disease.

Smoking cessation and inflammatory bowel disease.

Smoking and quit and Crohn’s disease.

Smoking and quit and inflammatory bowel disease.

Box 3. **Search query used to collect articles in Cochrane Library**

Smoking cessation and Crohn’s disease.

The lists of references in the included trials were also screened for relevant studies.

### Eligible studies for this review

Eligible studies were randomized control trials (RCT), prospective and historical cohort studies, published from inception to February 2022, written in English or French. Articles not peer reviewed, editorials, letter to editors, comments were excluded.

### Type of participants

We included published articles dealing with the effectiveness of SC interventions in patients with CD aged 16 years or higher, regardless of the setting in which the interventions were carried out (hospital, routine visits, work, home). The diagnosis of CD had to be confirmed on the basis of histological, endoscopic and radiological data. Regarding the smoking pattern, we have included studies in which patients had been smoking at least one cigarette per week. Studies that included former smokers were excluded. Studies that examined patients with mental disorders or learning disabilities were excluded as these groups are thought to have heavier and a more dependent smoking pattern and a less likeliness to quit.

### Type of interventions

We included interventions that primarily aimed to help CD patients, who are actively smoking or who present smoking relapses, to quit. The strategies ranged from cognitive and interventions that targeted life skills development to pharmacological approaches or a combination of different interventions. The interventions were categorized into the following three categories: pharmacological therapy, non pharmacological strategies and complex strategies associating both.

Pharmacological therapy included antidepressants drugs, mainly bupropion, nicotine receptor partial agonists and nicotine replacement therapy which can be delivered by the following systems: patch, spray, lozenge and inhaler. Nonpharmacological interventions include behavioral therapy, motivational interviewing, physician advice, telephone-based interventions. The motivational interviewing is a counselling approach that aims exploring patients uncertainties about modifying their behavioral and help them in resolve them. Behavioral interventions refer to approaches targeting the identification and modification of smoking associated behaviors.

### Type of outcome measure

We were interested in the percentage of patients who managed to achieve SC at least once during the follow-up period, whether or not it is prolonged (over a period of 6 months). A biochemically validated abstinence was preferred. Yet, as this variable may not be used in all studies, we considered both self-reported and biochemically proved SC.

To be able to compare the outcomes of the interventions in patients with CD to the general population, we have expressed the outcomes in studies with control groups as unadjusted odds ratios using the following formula:

$$\left[\frac{\left(\text{Number of quitters intervention}\right)}{\text{Number of continuing smokers intervention}}\right]\left[\frac{\left(\text{Number of quitters control}\right)}{\text{Number of continuing smokers control}}\right]$$

### Study identification & data extraction

The identified studies from the databases were initially reviewed for duplication. After duplicates removal, all abstracts were reviewed to exclude studies that do not meet our inclusion criteria. The remaining studies were systematically reviewed by two different authors. In cases of disagreement, a third author was consulted and the differences were resolved with a consensus based discussion.

The following data were extracted for each article the identification features (authors, article title, country of origin), study characteristics (aim of the study, study design, the setting of the intervention), characteristics of the participants (age, gender, comorbidities, the phenotype of CD, presence of perineal manifestations, prior surgeries for CD or complications such as abscess and ongoing medications), SC interventions that has been implemented (type frequency, setting and control(s) if applicable), outcomes and conclusions made by authors.

### Quality evaluation

The evaluation of these randomized controlled trials was made using the CONSORT guidelines in order to make a homogenization between different trials to improve their methodology and validation. Two independent reviewers performed the quality assessments. The outcomes were then compared and disagreements were resolved with consensus.

## Results

### Study of the included papers

**Figure 1.**
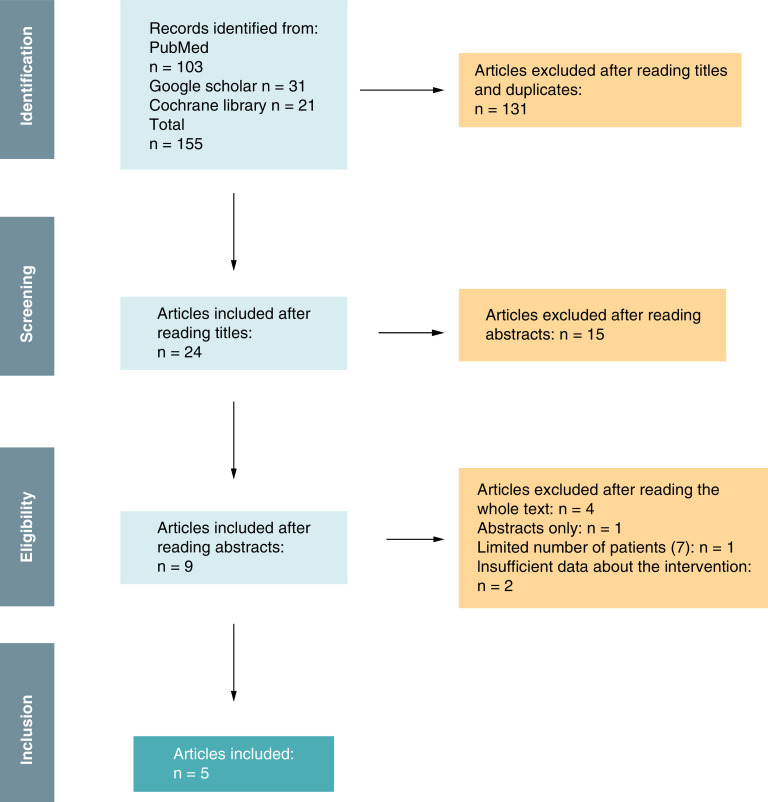
Flowchart of literature searching based on PRISMA guidelines.

A total of 155 articles were initially identified using the research query mentioned above (PubMed, Google Scholar). After reading titles and eliminating duplicates, we retained 24 articles. On the second selection and after reading abstracts and applying the inclusion and exclusion criteria, we retained nine articles that were fully assessed. Eventually, five articles met the research criteria and were included in the study (Figure 1). With the exception of Cosnes *et al.* study, all including studies were published between 2020 and 2022.

### Characteristics of studies

Table 1 depicts a summary of the studies included in the systematic review. Two studies were conducted in the USA [22,23], the remaining studies were conducted, respectively, in Italy, Spain and France [14,24,25]. Regarding studies design, two studies were RCT [14,23], the rest were cohort studies. In all five included articles, the study settings were IBDs clinical centers.

**Table 1.** Characteristics of included studies (n = 5).

Author and Journal	Groups: Definition and patients number	Type of intervention and frequency	Aims/outcomes	Outcome measure	Main results
Tse *et al.* USA Single-center prospective cohort	Intervention group: n = 19 Patients who agreed to participate in at least one session of the program Control group: n = 13 Patients who did participate in any sessions	Motivational interviewing + behavioral counseling + pharmacologic management	**Primary outcomes:** Proportion of patients who quit smoking for 30 days or more at least once **Secondary outcome:** Proportion of continued quitters, relapses, and increased or decreased daily cigarette number in both groups	Self-reported smoking abstinence + Time Smoke-Free of at least 30 days	SC proportions: 42% of the enrolled patients 15% of the declined patients
Correal *et al.* Spain RCT	Patients included were randomized to: The intervention group (n = 67) The control group (n = 68)	The 5 R's motivational interviewing every 3 months for at least 12 months.	**Primary outcomes:** changes in the motivation to quit smoking after 12 months **Secondary outcome:** the number of patients who achieve SC in the intervention and control groups	Self-reported smoking abstinence	No statistically significant difference was found Between the intervention and control regarding SC rate (p < 0.237) 20.9% of the intervention group 13.2% of the control group
Santus *et al.* Italy Historical corhort	All patients with CD included underwent the intervention (n = 27)	Motivational and group psychological counselling each week the first month, +/-Pharamcological therapy (varenicline or nicotine replacement therapy)	Compare the effectiveness of a multidisciplinary SC program in patients with and without CD. Identify factors of quitting failure in patients with CD.	exhaled CO value (≤6.5)	14.8% of patients with CD quit smoking The SC rate was significantly lower in patients with CD when compared with subjects without CD. (14.8 vs 36.7%, p < 0.022) Compared with other participants, smokers with CD have higher rates of anxiety and depression.
Scoville *et al.* USA RCT	Usual care group (n = 14) motivational interviewing, referral to a free telephone cessation line (Quitline) non-NMR based pharmacological therapy Metabolism-informed care: (n = 17) motivational interviewing, referral to a free telephone cessation line (Quitline) patients were informed with their NMR and could choose an NMR based pharmacological therapy	Patient's choice of pharmacotherapy was guided by the nicotine metabolite ratio Slow metabolizers: NRT Normal metabolizers: non-nicotine based pharmacotherapy	Aim: Determine the prevalence of smoking in a CD population Evaluate smokers' attitudes toward a strategy based on individual assessment of nicotine metabolism Outcomes: Primary outcomes Intervention satisfaction and match rates between NMR and medication choice. Secondary outcomes combined end point: ≥50% decrease in cigarettes smoked per day or complete cessation at 6 months	7-day point-prevalence abstinence and/or cigarette consumption	The rate of patients choosing a medication matching their NMR was higher in the the MIC group (100 vs 64%, p = 0.01) 16.1% quit smoking 48.4% reached the combined end point No statistical significance difference in the combined end point between the two groups
Cosnes *et al.* France Prospective cohort	All patients with CD included underwent the intervention (n = 474)	repeatedly given behavioral counseling for patients + mean daily cigarettes >15: weekly visits with a specialized physician +/- NRT and fluoxetine	Aim: evaluate the benefit of SC Primary end point: the flare-up rate and time to flare-up SC	urinary cotinine on an aliquot	25% of patients: SC for more than 15 days 12% remained abstinent >12 months Quitters had a significantly lower risk of flare-up when compared with active smokers (p < 0.001).

CD: Crohn’s disease; CO: Carbon monoxide; NRT: Nicotine metabolite ratio; RCT: Randomized controlled trial; SC: Smoking cessation.

### Characteristics of participants

The study sample sized ranged from 17 to 474 patients. In Cosnes *et al.* study, patients with malignancies and irregular follow-up were excluded. In all remaining studies no eligibility criteria regarding CD extension, severity or related complications were applied. In one study, patients had to present no contraindications to taking two out of the three SC medications to be included [23]. On the other hand, in Correal *et al.*’s RCT, all patients included had to have internet access and an e-mail account [14]. The mean age of patients ranged from 49 to 55 years. In one study, a male predominance was noted (58%, respectively) [24] while in the remaining four studies most of patients included were female [14,22,23,25]. In three studies, patients educational levels were detailed [14,23,24]. The rate of college graduates ranged from 8 to 52.9%. Data regarding CD phenotype and location were mentioned in three studies [14,22,25]. In Correal *et al.*’s study, ilecolic CD was the most common disease location among the intervention group (44.4%) and non structuring non penetrating CD was the predominant phenotype while perianal CD was found in 18% of cases [14]. Similarly in Tse *et al.* study, the predominant disease location and phenotype were ileocolonic CD and nonpenetrating nonstructuring disease [22]. One study used The Harvey Bradshaw Index to assess CD activity [22]. In the remaining studies, CD activity was not evaluated. The medication used for CD were detailed in three studies [14,22,23]. The rate of patients on steroids undergoing the interventions ranged from 4.2 to 11.8% while the rate of those on biological agents ranged from 54.2 to 94.1%. None of the studies included reported CD-related complications while the rate of extraintestinal manifestations was reported in only one study (30.5% of the intervention group) [14].

The instruments used to describe smoking characteristics differed across the articles. Two studies determined the patients’ daily cigarettes with respective median number of 10 and 17.5 [14,24]. In Cosnes *et al.* study, patients were divided to ranges according to mean smoked cigarettes with 38% of them consuming more than 20 cigarettes per day [25]. Tse *et al.* quantified smoking in pack-year [22] while Santus *et al.* used both [24]. Nicotine dependence was assessed in two studies with The Heaviness Smoking Index and the Fagerström Test, respectively. The motivation to quit was assessed with the Richmond test in Correal *et al.*’s study and with the motivation to quit score by Santus *et al.* [14,24]. Three studies assessed previous attempts to quit smoking [14,22,24]. The median of attempts number was two in two studies [14,22]. The third study determined the percentage of patients who had attempted to quit (33.3%) [24].

### Initial motivation

The initial motivational level of patients was assessed in three studies [14,22,24]. For instance, Correal *et al.* included patients who refused referral to a SC Unit after receiving the 5A’s intervention. In addition, when assessing motivation with Richmond test in this study, nearly half of patients in the intervention group were found to have a low motivation level [14]. On the other hand, in Tse *et al.* study, all the included patients were interested in the SC program [22]. In Santus *et al.* study, all patients included had already been referred to a SC program [24]. The median value of the motivation to quit score among patients undergoing the intervention was 10 with an IQR of [9–13].

### Nature of interventions

#### Motivational intervention

Motivational interviewing was used in four studies [14,22–24]. In three studies, it was associated with other interventions [22–24] and it was exclusive and based on telephone conversations in Correal *et al.*’s study [14]. Details concerning the motivational intervention were only reported in two studies [14,22]. In both cases, the theoretical framework used was the WHO 5Rs (Relevance, Risks, Rewards, Roadblocks, and Repetition). The intervention duration ranges from 5 to 10 min and comprises five steps that aim to repeatedly do the following: help the patient to understand why SC is personally relevant, assist the patient in identifying harmful effects of smoking and benefits of SC and finally help the patient to determine barriers to quitting. In Tse *et al.*’s study, motivational interviewing was provided face-to-face during the initial visit. On the other hand, the intervention in Correal *et al.*’s study was an exclusive 5R motivational interviewing received every 3 months for over 12 months by phone conversations.

#### Psychological & behavioral counseling

Counseling sessions were used in four studies through different strategies and with different frequency and duration. In one study, patients attended group psychological counselling each week during the first 4 weeks [24]. In Tse *et al.’*s study, patients included were offered behavioral counselling, yet no details concerning the number of sessions and their duration were provided [24]. In another study, patients were referred to a Quitline which is an evidence-based SC intervention where counseling is provided by highly trained healthcare professionals. Yet, the number of patients who did contact the Quitline and used its service was not mentioned [23]. In Cosnos *et al.* study, patients were offered behavioral counselling or, for those smoking than 15 cigarettes per day, referral to a SC program where they can visit a specialized physician weekly and potentially benefit from SC pharmacotherapy [25].

#### Pharmacological therapy

Pharmacological therapy was used in four studies [22–25]. In Scoville *et al.’*s study, SC medication were prescribed in all cases and patients could choose among pharmacological agents either with or without information provided regarding the nicotine metabolite ratio [23]. In Tse *et al.* study, nicotine patch combined with nicotine gum or lozenge were prescribed for individuals smoking ten cigarettes per day or more or in cases where the latter two failed in monotherapy [22]. On the other hand, in Santus *et al.* and Cosnes *et al.* studies, the criteria based on which patients were prescribed pharmacological agents were not detailed [24].

#### Outcome measures

Several operational definitions of smoking were used in the included studies. In Correal *et al.* study, patients included smoked at least seven cigarettes per week for at least 6 months. In Tse *et al.* study, the included patients smoked every day during the 30 days prior to inclusion [22]. In Scoville and Cosnos studies, the inclusion criteria included a cutoff of, respectively, five and three cigarettes per day [23]. Finally, smoking status was not defined in Santus *et al.’*s study as the authors opted for not applying any strict eligibility criteria in order to reflect the real world practice [24]. Among the studies included, only two did confirm self-reported abstinence with biochemical methods with measurement of the concentration of exhaled carbon monoxide (CO) and urinary cotinine, respectively [24,25].

Operational definitions of smoking, SC, outcomes and odds ratio are summarized in Table 2.

**Table 2.** Definitions of reported smoking and smoking cessations and extracted odds ratios.

Authors	Definition of smoking	Definition of smoking abstinence	Odds ratio
Correal *et al.*	Self-reported smoking of more than seven cigarettes/week for at least 6 months	Self-reported smoking abstinence	1.73
Tse *et al.*	Self-reported smoking every day during the 30 days prior to inclusion	Time smoke-free of at least 30 days	3.99
Santus *et al.*	–	Exhaled CO value (≤6.5)	–
Scoville *et al.*	Self-reported smoking of at least five cigarettes per day	A 7-day point-prevalence abstinence and/or cigarette consumption	–
Cosnes *et al.*	Self-reported smoking of at least three cigarettes per day	Determination of urinary cotinine on an aliquot	–

#### Effects of interventions

In Tse *et al.* study, the rate of patients in the intervention group who did quit smoking was 42 versus 15% in the intervention group with an OR of 3.99 [22]. Smoking abstinence rate was also higher in the intervention group in Correal *et al.* study (20.9 vs 13.2%, p < 0.237). The difference was not statistically significant [14]. In Santus and Cosnes studies, 14.8 and 25% of patients with CD did quit smoking, the OR could not be calculated as all patients with CD underwent the interventions [24]. In Scoville *et al.* study, outcomes regarding SC were measured with an end point that combined either complete smoking abstinence at 6 months or a decrease by at least 50% in the number of daily cigarettes. The results indicated no statistically significant difference between the two groups [23]. Finally, the effect of the interventions on disease activity was analyzed only by Cosnes *et al.* with the study finding a lower risk of flare-up in quitters when compared with active smokers (p < 0.001).

#### Methodological quality

Evaluation of the included RCT with the CONSORT checklist indicated a moderate quality in Scoville study due to the lack of a predetermined sample size and the absence of blinding interventions. On the other hand, Correal *et al.* study complied with the CONSORT checklist.

## Discussion

In this study, we identified two RCTs and three cohort studies of SC interventions in patients with CD. We eliminated two eligible studies as the interventions were not sufficiently detailed to allow replication in two studies. At the outcome level, The SC rates ranged from 14.8 to 42% and was ≤25% in four studies. The higher rate was observed in Tse *et al.*’ study [14]. It is worth mentioning though that this study had the lowest threshold of cigarettes number to define smoking which may indicate a lower nicotine dependence level. A similar high SC rate was previously reported in the TABCROHN study by nst *et al.* (31%) [26]. The study was not included in this systematic review as different SC strategies were used according to each center practice and available resources. Overall, pharmacological agents were received by only 12% and yet, the overall SC rate was 31%. The differences in SC rates between the included studies can be explained by several factors including a wide range of operational definitions of smoking and quitting with outcome measure relying on an evaluation of a point prevalence in some studies and continuous SC or both in others. In addition, only two studies validated self-reported SC with biochemical methods [24,25]. Nicotine dependence and patients’ motivation differed as well between studies. In addition, the sample size varied widely between the studies. In three studies, the number of CD patients undergoing the interventions was lower than 30 [22–24] while Cosnes study included over 400 patients [25]. These factors make it difficult to compare the effectiveness of the programs used in the included studies. On the other hand, the success rates of SC programs in the general population are reported to range from 22 to 45% [27]. In Santus study, the same SC program was found to be significantly more efficacious in patients without CD and the low quit rate was partially attributed to a high prevalence of anxiety and depression in the included patients CD patients which resulted in low motivation to quit [24]. The low success rate of SC interventions can also be explained by low adherence to pharmacological agents, a common observation in patients with IBD, especially the young adults. Jackson *et al.* found in a systematic review high non-adherence to medication rates in patients with IBD with most studies reporting rates as high as 30–45% [28,29]. The fact that the pharmacological agents were not covered by health insurance and were not provided by the SC program in Santus study could have contributed to patients noncompliance. These speculations cannot be confirmed or denied as none of the studies included evaluated adherence rates. It is worth mentioning though that a recent systematic review evaluating SC interventions in patients with chronic diseases, who are also exposed to a high medication burden, found that in six out of the ten included articles, patients in the intervention groups had significantly higher abstinence rates [30]. Hence, polypharmacy do not necessarily result in low adherence and low SC rates if the patients are integrated in an efficacious intervention. Regarding the mean of delivering counseling, it has been suggested that effective interventions rely on face-face sessions to construct and foster a relationship with the patient [30]. In patients with CD, the same strategy did not yield as high SC rates while Correal telephone based program resulted in more promising results. Overall, predictive factors of SC failure in CD patients undergoing specific interventions remain mostly undefined. Cosnes *et al.* found that demographical features, CD and smoking duration did not significantly affect outcomes [25]. Initially, the TABACROHN study highlighted the importance of repetitive counselling and patient–doctor relationship as high rates of SC were found in spite of inhomogeneous interventions. Further support for this theory comes from the results of Ho *et al.* systematic review who found that in patients with chronic diseases, it is the programs with intensive regular counseling sessions that result in better SC rates [30]. Yet, the results of more recent studies do not corroborate this theory, as interventions with multiple counseling sessions did not necessarily lead to high SC rates [24].

To sum, in spite of substantial effect of tobacco smoking on CD course, evidence regarding the effectiveness of SC programs in this population is very limited in number and in sample size which makes it difficult to draw conclusions regarding the interventions, the theoretical frameworks and the int1erventionists. Overall, SC programs evaluated seem to be less effective in CD patients than in the general population and the reasons for this are not well known. Telephone based counseling do not seem to be less effective and might be the solution to overcome the shortness in healthcare resources especially in low-to middle income countries.

In order to properly evaluate SC interventions, we suggest the following recommendations. First, standardizing definitions of smoking and smoking abstinence with use of biochemical methods to verify self-reported abstinence. Second, referral to a psychologist may be considered to screen patients for anxiety or depression symptoms seen the high rates of both in this population. Third, physicians should assess medication adherence and ideally, SC programs should provide pharmacological therapy for SC to patients to limit compliance issues. Fourth, intensive regular counseling, whether delivered by phone or by face-to-face sessions is essential and should be a part of any SC program.

## Conclusion

The findings of this systematic review do not allow us to conclude to a higher efficacy of any particular form of behavioral or pharmacological therapy. We believe that implementation and evaluation of tailored SC interventions for CD patients must be promptly addressed in further studies.

## Future perspective

As further research continues to unravel the complex relationship between smoking and CD pathogenesis, novel strategies and interventions should be developed to effectively target SC in this patient population. With the growing recognition of the detrimental effects of smoking on CD outcomes, healthcare providers and researchers may explore innovative approaches, such as tailored behavioral interventions, personalized medicine and telehealth interventions, to improve SC rates among patients with CD. Furthermore, leveraging technological advancements and digital health tools, such as mobile apps and wearable devices, may provide additional opportunities for monitoring and supporting SC efforts in real-time. As our understanding of the unique challenges and barriers to smoking cessation in CD patients deepens, future research may also uncover ways to optimize intervention delivery, address psychosocial factors and improve long-term quit rates. Overall, continued efforts to develop evidence-based SC interventions that are specifically tailored to patients with CD have the potential to significantly improve patient outcomes, reduce disease burden and ultimately enhance the overall health and well-being of individuals living with this chronic inflammatory condition.

**Summary points**

- Two randomized controlled trials (RCTs) and three cohort studies were identified for smoking cessation (SC) interventions in patients with Crohn’s disease (CD).
- SC rates ranged from 14.8 to 42% with most studies showing rates of ≤25%.
- Variability in operational definitions of smoking and quitting, outcome measures, nicotine dependence, motivation and sample sizes among studies made it difficult to compare the effectiveness of interventions.
- Factors such as low medication adherence, high rates of anxiety and depression in CD patients, and lack of coverage for pharmacological therapy may contribute to low SC rates.
- Standardization of definitions, use of biochemical methods for verifying abstinence, referral for psychological screening, assessment of medication adherence and intensive counseling may be recommended for evaluating SC interventions in CD patients.

## References

[1] To N, Gracie DJ, Ford AC. Systematic review with meta-analysis: the adverse effects of tobacco smoking on the natural history of Crohn's disease. Aliment. Pharmacol. Ther. 43, 549–561 (2016). 26749371 10.1111/apt.13511

[2] Kaplan GG, Windsor JW. The four epidemiological stages in the global evolution of inflammatory bowel disease. Nat. Rev. Gastroenterol. Hepatol. 18, 56–66 (2021). 33033392 10.1038/s41575-020-00360-x PMC7542092

[3] Kappelman MD, Rifas-Shiman SL, Kleinman K The prevalence and geographic distribution of Crohn's disease and ulcerative colitis in the United States. Clin. Gastroenterol. Hepatol. 5, 1424–1429 (2007). 17904915 10.1016/j.cgh.2007.07.012

[4] Floyd DN, Langham S, Séverac HC, Levesque BG. The economic and quality-of-life burden of Crohn's disease in Europe and the United States, 2000 to 2013: a systematic review. Dig. Dis. Sci. 60, 299–312 (2015). 25258034 10.1007/s10620-014-3368-z

[5] Knowles SR, Graff LA, Wilding H, Hewitt C, Keefer L, Mikocka-Walus A. Quality of life in inflammatory bowel disease: a systematic review and meta-analyses – part I. Inflamm. Bowel Dis. 24, 742–751 (2016). 10.1093/ibd/izx100 29562277

[6] van der Have M, van der Aalst KS, Kaptein AA Determinants of health-related quality of life in Crohn's disease: a systematic review and meta-analysis. J. Crohns Colitis 8, 93–106 (2014). 23746864 10.1016/j.crohns.2013.04.007

[7] Park KT, Ehrlich OG, Allen JI The cost of inflammatory bowel disease: an initiative from the Crohn's & Colitis Foundation. Inflamm. Bowel Dis. 26, 1–10 (2020). 31112238 10.1093/ibd/izz104 PMC7534391

[8] Yu AP, Cabanilla LA, Wu EQ, Mulani PM, Chao J. The costs of Crohn's disease in the United States and other Western countries: a systematic review. Curr. Med. Res. Opin. 24, 319–328 (2008). 18067689 10.1185/030079908x260790

[9] Bassi A, Dodd S, Williamson P, Bodger K. Cost of illness of inflammatory bowel disease in the UK: a single centre retrospective study. Gut 53, 1471–1478 (2004). 15361497 10.1136/gut.2004.041616 PMC1774248

[10] Zhao M, Gönczi L, Lakatos PL, Burisch J. The burden of inflammatory bowel disease in Europe in 2020. J. Crohns Colitis. 15, 1573–1587 (2021). 33582812 10.1093/ecco-jcc/jjab029

[11] Xavier RJ, Podolsky DK. Unravelling the pathogenesis of inflammatory bowel disease. Nature 448, 427–434 (2007). 17653185 10.1038/nature06005

[12] Jairath V, Feagan BG. Global burden of inflammatory bowel disease. Lancet Gastroenterol. Hepatol. 5, 2–3 (2020). 31648974 10.1016/S2468-1253(19)30358-9

[13] Nunes T, Etchevers MJ, Domènech E Smoking does influence disease behaviour and impacts the need for therapy in Crohn's disease in the biologic era. Aliment. Pharmacol. Ther. 38(7), 752–760 (2013). 23980933 10.1111/apt.12440

[14] Navarro Correal E, Casellas Jorda F, Borruel Sainz N Effectiveness of a telephone-based motivational intervention for smoking cessation in patients with Crohn disease: a randomized, open-label, controlled clinical trial. Gastroenterol. Nurs. 44, 418–425 (2021). 34269705 10.1097/SGA.0000000000000572 PMC8635256

[15] Nunes T, Etchevers MJ, García-Sánchez V Impact of smoking cessation on the clinical course of Crohn's disease under current therapeutic algorithms: a multicenter prospective study. Am. J. Gastroenterol. 111, 411–419 (2016). 26856753 10.1038/ajg.2015.401

[16] Lunney PC, Kariyawasam VC, Wang RR Smoking prevalence and its influence on disease course and surgery in Crohn's disease and ulcerative colitis. Aliment. Pharmacol. Ther. 42, 61–70 (2015). 25968332 10.1111/apt.13239

[17] Leung Y, Kaplan GG, Rioux KP Assessment of variables associated with smoking cessation in Crohn's disease. Dig. Dis. Sci. 57, 1026–1032 (2012). 22311366 10.1007/s10620-012-2038-2

[18] Dziekiewicz M, Kowalska-Duplaga K, Baranowska-Nowak M Awareness of smoking in adolescents with inflammatory bowel disease. Ann. Agric. Environ. Med. 27, 61–65 (2020). 32208581 10.26444/aaem/105821

[19] Le Berre C, Loy L, Lönnfors S, Avedano L, Piovani D. Patients' perspectives on smoking and inflammatory bowel disease: an online survey in collaboration with European Federation of Crohn's and Ulcerative Colitis Associations. World J. Gastroenterol. 26, 4343–4355 (2020). 32848338 10.3748/wjg.v26.i29.4343 PMC7422536

[20] Ryan WR, Ley C, Allan RN, Keighley MRB. Patients with Crohn's disease are unaware of the risks that smoking has on their disease. J. Gastrointest. Surg. 7, 706–711 (2003). 12850686 10.1016/s1091-255x(03)00066-0

[21] Babb S, Malarcher A, Schauer G, Asman K, Jamal A. Quitting smoking among adults - United States, 2000-2015. Morb. Mortal. Wkly Rep. 65, 1457–1464 (2017). 10.15585/mmwr.mm6552a1 28056007

[22] Tse SS, Sands BE, Keefer L Improved smoking cessation rates in a pharmacist-led program embedded in an inflammatory bowel disease specialty medical home. J. Pharm. Pract. 35(6), 827–835 (2021). 33827316 10.1177/08971900211000682

[23] Scoville EA, Tindle HA, Wells QS Precision nicotine metabolism-informed care for smoking cessation in Crohn's disease: a pilot study. PLOS One 15, e0230656 (2020). 32214373 10.1371/journal.pone.0230656 PMC7098646

[24] Santus P, Radovanovic D, Raiteri D The effect of a multidisciplinary approach for smoking cessation in patients with Crohn's disease: results from an observational cohort study. Tob. Induc. Dis. 18, 29 (2020). 32336967 10.18332/tid/119161 PMC7177387

[25] Cosnes J, Beaugerie L, Carbonnel F, Gendre J-P. Smoking cessation and the course of Crohn's disease: an intervention study. Gastroenterology 120, 1093–1099 (2001). 11266373 10.1053/gast.2001.23231

[26] Nunes T, Etchevers MJ, Merino O High smoking cessation rate in Crohn's disease patients after physician advice--the TABACROHN Study. J. Crohns Colitis 7, 202–207 (2013). 22626507 10.1016/j.crohns.2012.04.011

[27] Yilmazel Ucar E, Araz O, Yilmaz N Effectiveness of pharmacologic therapies on smoking cessation success: three years results of a smoking cessation clinic. Multidiscip. Respir. Med. 9, 9 (2014). 24495744 10.1186/2049-6958-9-9 PMC3916028

[28] Jackson CA, Clatworthy J, Robinson A, Horne R. Factors associated with non-adherence to oral medication for inflammatory bowel disease: a systematic review. Am. J. Gastroenterol. 105, 525–539 (2010). 19997092 10.1038/ajg.2009.685

[29] Lenti MV, Selinger CP. Medication non-adherence in adult patients affected by inflammatory bowel disease: a critical review and update of the determining factors, consequences and possible interventions. Expert Rev. Gastroenterol. Hepatol. 11, 215–226 (2017). 28099821 10.1080/17474124.2017.1284587

[30] Ho LLK, Li WHC, Cheung AT, Xia W. Effectiveness of smoking cessation interventions for smokers with chronic diseases: a systematic review. J. Adv. Nurs. 77, 3331–3342 (2021). 33896036 10.1111/jan.14869

